# Effects of short- and long-term glucocorticoid-induced osteoporosis on plasma metabolome and lipidome of ovariectomized sheep

**DOI:** 10.1186/s12891-020-03362-7

**Published:** 2020-06-05

**Authors:** Diana Cabrera, Marlena Kruger, Frances M. Wolber, Nicole C. Roy, Karl Fraser

**Affiliations:** 1 Food Nutrition & Health Team, AgResearch Grasslands, Tennent Drive, Palmerston North, 4442 New Zealand; 2 School of Health Sciences, Massey University, Tennent Drive, Palmerston North, 4442 New Zealand; 3 Riddet Institute, Massey University, Palmerston North, 4442 New Zealand; 4 School of Food Advanced technology, Massey University, Tennent Drive, Palmerston North, 4442 New Zealand; 5 Centre for Metabolic Health Research, Massey University, Tennent Drive, Palmerston North, 4442 New Zealand; 6 High-Value Nutrition National Science Challenge, Auckland, 1142 New Zealand

**Keywords:** Osteoporosis, Metabolome, Lipidome, OVX sheep

## Abstract

**Background:**

Understanding the metabolic and lipidomic changes that accompany bone loss in osteoporosis might provide insights about the mechanisms behind molecular changes and facilitate developing new drugs or nutritional strategies for osteoporosis prevention. This study aimed to examine the effects of short- or long-term glucocorticoid-induced osteoporosis on plasma metabolites and lipids of ovariectomized (OVX) sheep.

**Methods:**

Twenty-eight aged ewes were divided randomly into four groups: an OVX group, OVX in combination with glucocorticoids for two months (OVXG2), and OVX in combination with five doses of glucocorticoids (OVXG5) to induce bone loss, and a control group. Liquid chromatography–mass spectrometry untargeted metabolomic analysis was applied to monthly plasma samples to follow the progression of osteoporosis over five months.

**Results:**

The metabolite profiles revealed significant differences in the plasma metabolome of OVX sheep and OVXG when compared with the control group by univariate analysis. Nine metabolites were altered, namely 5-methoxytryptophan, valine, methionine, tryptophan, glutaric acid, 2-pyrrolidone-5-carboxylic acid, indole-3-carboxaldehyde, 5-hydroxylysine and malic acid. Similarly, fifteen lipids were perturbed from multiple lipid classes such as lysophoslipids, phospholipids and ceramides.

**Conclusion:**

This study showed that OVX and glucocorticoid interventions altered the metabolite and lipid profiles of sheep, suggesting that amino acid and lipid metabolisms are potentially the main perturbed metabolic pathways regulating bone loss in OVX sheep.

## Background

Osteoporosis cases are most common in the elderly population, with postmenopausal women the most affected as estrogen withdrawal induces bone loss. Currently, the diagnosis of osteoporosis is carried out by measuring bone mineral density (BMD) as well as measuring bone turnover biomarkers [1–4]. While bone imaging scans are accepted as non-invasive methods, changes in BMD can only be detected by this method over a period of years [3, 4]. Thus, the development of a fast and simple prognostic method is desired, where the detection of early biomarkers for osteoporosis can be used to diagnose or predict the risk of bone loss in postmenopausal women.

Metabolomics is an emerging tool for studying many diseases including cancer, diabetes, Alzheimer’s, and osteoarthritis disease [5–7]. Metabolomics allows the measurement and characterization of small molecules such as organic and amino acids, saccharides, lipids and other biomarkers in a biological system [8]. The metabolome is highly dynamic, and changes can occur rapidly in response to physiological conditions or treatments. These metabolic alterations may have the potential for enabling early detection of changes of metabolic pathways and discovering novel biomarkers that can be associated with human diseases [9–12].

Lipidomics is a sub-analysis of the metabolome, which studies the characterization of lipid molecular species [13]. Lipid metabolism alterations in bone, specifically in the cellular balances between adipocytogenesis and osteoblastogenesis in the bone marrow, are controlled by many factors. During menopause, lipid accumulation alters bone marrow cells and could be a determinant of increased bone remodelling that may affect bone mass, and has been associated with obesity and osteoporosis in postmenopausal women [14, 15]. Lipids and their derivatives, such as sphingosine-1-phosphate, lysophosphatidic acid and some fatty acid amides, are known to regulate cellular process, and any change in these can lead to bone pathologies such as osteoporosis [16]. Metabolomics and lipidomics have been used in both animal models and postmenopausal women with low BMD or osteoporosis. Evidence suggests that perturbations in metabolic pathways such as the TCA cycle, glutamine metabolism, amino acid metabolism and fatty acid metabolism due to estrogen deficiency might impact bone remodelling in ovariectomized (OVX) rats and mice [17–23]. However, the clinical symptoms in these rodent models are poorly representative of the naturally occurring postmenopausal condition in humans [24].

Sheep are accepted as one of the large animal models for osteoporosis. Most of the preclinical and translational studies have reported effective methods for inducing bone loss in sheep through OVX alone or by the combined treatment of OVX, a calcium- and vitamin D-deficient diet, and glucocorticoid treatments [25]. Glucocorticoids affect bone remodelling by inducing increased osteoclast bone resorption and reducing osteoblast formation; they also perturb calcium metabolism by inhibiting its absorption, which leads to a calcium deficit and an altered mineral metabolism [26–28]. Previous studies on sheep have documented the mechanisms underlying bone loss in postmenopausal osteoporosis [29–31], but no research has been conducted to evaluate whether OVX alone or when combined with glucocorticoid interventions affect the plasma metabolite and lipid profiles of OVX sheep. Thus, it was hypothesized that untargeted metabolomics can be used to unravel the underlying metabolic changes in plasma of OVX sheep treated with glucocorticoids, and the biochemical changes that will be associated with bone loss.

The purpose of this study was to determine the short and long-term impact of estrogen deficiency and glucocorticoid hormonal interventions on plasma metabolism of OVX sheep, an animal model for human osteoporosis, using a liquid chromatography–mass spectrometry (LC–MS) metabolomics approach.

## Methods

Animals and experimental design have been described elsewhere by Cabrera et al. [32]. Briefly, 28 Merino sheep (ewes) aged between 7 and 9 years were obtained from a commercial farm in the Whanganui region, New Zealand. Ewes were either untreated, or with hormonal interventions resulting from either OVX or OVX combined with methylprednisolone treatment (400 mg; Vetacortyl®, Vetoquinol SA, Lure Cedex, France) (OVXG). For the short-term study the study treatments were: untreated control group *n* = 10, OVX group *n* = 12 and OVXG *n* = 6 for two months. After two months, half of the ewes in the control group and OVX group were randomly sacrificed using a captive bolt followed by exsanguination, and half of the ewes in the OVXG were assigned into either a group that received glucocorticoids for five months (OVXG5) or no glucocorticoid for the next three months (OVXG2). Then, for the long-term approach, the study treatments were: untreated control group *n* = 5, OVX group *n* = 6, OVXG2 *n* = 3, OVXG5 *n* = 3 from baseline (month zero) to five months. At the end of the study, the remaining ewes from all groups, were similarly euthanized. This study was performed in full compliance with the Massey University Animal Ethics committee (approval number 14/103) and performed according to the Code of Ethical Conduct for the use of live animals for research at Massey University, Palmerston North, New Zealand.

### Blood collection

Blood samples were taken from the 28 ewes at baseline (pre-surgery), and through the period of this study at one, two, three, four and five months post-surgery. The sheep were given ad libitum water and straw, and a measured amount of a standard pelleted diet twice daily. Blood was collected by venepuncture of the jugular vein and the flow directed into EDTA-treated plasma separator vacuum tubes. After centrifugation (2000 *g*, 15 min, 4 °C), the supernatant (plasma) was removed and stored (− 80 °C) until required.

### Chemicals

All standards and reagents used were analytical grade and supplied by Sigma-Aldrich Chemicals Co. (St Louis, MO, USA) unless specified. Ultrapure water was used for preparation of chemicals (Milli-Q-system, Millipore, Bedford, MA, USA). Acetonitrile (ACN), methanol and isopropanol were optima LC–MS grade, chloroform was high-performance liquid chromatography (HPLC) grade, and all were purchased from Thermo Fisher Scientific (Auckland, New Zealand). Ammonium formate and formic acid were purchased from Sigma–Aldrich Chemicals Co. (St Louis, MO). 16:0 *d*31–18:1 PE (phosphatidylethanolamine) was purchased from Avanti Polar Lipids, INC. (Alabaster, AL, USA).

### Metabolomic analysis

Methods for metabolite extraction were as have been described previously [33]. Metabolite extraction was performed using precooled chloroform:methanol (1:1 v/v, containing 0.8 mg mL^− 1^ of internal standards, *d*_4_-citric acid, ^13^C_2_-D-glucose, *d*_5_-L-tryptophan, *d*_7_-L-alanine, *d*_35_-stearic acid, *d*_5_-benzoic acid, *d*_10_-leucine, *d*_2_-tyrosine). Briefly, 100 μL of plasma was transferred to a microcentrifuge tube and 800 *μ*L of precooled chloroform:methanol at − 20 °C was added. The mixture was mixed by hand for 1 min and the samples incubated for 30 min at − 20 °C. Then, 400 μL of water was added to each sample, vortexed for 30 s and then centrifuged for 15 min at 11,000 rpm at 4 °C. Thereafter, 250 μL aliquots of the supernatant and 200 μL of the bottom layer were transferred into new microcentrifuge tubes for metabolite and lipid analyses respectively and evaporated to dryness under a stream of nitrogen.

The extraction residues were dissolved in 300 μL of ACN:water (1:1 v/v) containing formic acid (0.1%) for the metabolite analysis and 100 μL of Folch solvent mixture (chloroform:methanol 2:1 v/v) containing 16:0 *d*_31_–18:1-phosphatidylethanolamine internal standard at 10 μg mL^− 1^ concentration for the lipid analysis. The extracts were vortexed for 1 min, centrifuged for 10 min at 11,000 rpm at 4 °C, and 100 μL was transferred to a vial containing a limited volume insert and stored at 4 °C for immediate metabolite analysis. Blank samples were prepared using the same procedure but with 100 μL of water instead of plasma. The pooled quality control (QC) samples were prepared by adding 60 μL of each extract to form a QC stock. Then aliquots of 100 μL of the pooled QC sample were transferred into multiple pooled QC vials, evaporated to dryness and dissolved in the same manner as the rest of the samples.

LC–MS analysis was performed using a Thermo Exactive LC–MS system (Thermo Fisher Scientific, Waltham, MA, USA) consisting of an Accela 1250 quaternary pump, a Thermo-PAL autosampler fitted with a 15,000 psi injection valve (CTC Analytics AG., Zwingen, Switzerland) and a 2 μL injection loop and an Orbitrap mass spectrometer.

Metabolites were separated on a ZIC-pHILIC column (100 mm × 2.1 mm, 5 μm; Merck, Darmstadt, Germany) with a gradient elution program at a flow rate of 250 μL min^− 1^ as previously published [34]. The mobile phase was a mixture of ACN:formic acid (99.9:0.1 v/v) (solvent A) and water–ammonium formate (16 mM, pH 6.3) (solvent B). Data were collected in profile data acquisition mode over a mass range of *m/z* 55–1100 at a mass resolution setting of 25,000 with a maximum trap fill time of 100 ms using the Xcalibur v2.1 software (Thermo Scientific, Hemel Hemstead, UK) provided by the manufacturer.

Chromatographic separation of lipid extracts was conducted using a CSH-C18 column (100 × 2.1 mm; 1.7 μm particle size, Waters, Milford, MA, USA). The gradient elution was performed at a flow rate of 600 μL/min as previously published [35]. The analysis was performed using two solvents: solvent A was a mixture of 0.1% formic acid in ACN–water (60:40 v/v) with 10 mM ammonium formate, and solvent B was a mixture of 0.1% formic acid in isopropanol–ACN (90:10 v/v) with 10 mM ammonium formate (solvent B). Data were collected in profile data acquisition mode over a mass range of *m/z* 200–2000 at a mass resolution setting of 70,000 with a maximum trap fill time of 100 ms using the Xcalibur v2.1 software (Thermo Fisher Scientific, USA). The parameter settings for MS^2^ analysis were a mass resolution of 35,000 and a maximum trap fill time of 250 ms.

Each metabolite and lipid analysis was run in both positive and negative ionization mode separately. Pooled QC samples of the lipid extract were also rerun in data-dependant MS^2^ mode to collect fragmentation data for lipid annotation using LipidSearch™ (Thermo Fisher Scientific, USA) and LipidBlast software package (http://fiehnlab.ucdavis.edu/projects/LipidBlast). Metabolites were identified by matching accurate mass *m/z* and retention time to an in-house library and where no match existed, *m/z* was searched using the public databases HMDB (http://www.hmdb.ca/) and METLIN (https://metlin.scripps.edu/) with a 5 ppm mass accuracy window.

The resulting LC-MS files were converted to mzXML using the open source ProteoWizard converters (http://proteowizard.sourceforge.net/) and subsequent peak picking, alignment and integration of the LC-MS data set from the plasma metabolites and lipids were performed with the open-source software XCMS (Table S1 in Supplementary Materials). Analysis run-order effects were removed using the QC-based loess normalization in the Workflow4Metabolomics Galaxy workflow tool [36] and features detected in the blanks at the same abundance as the QC samples were removed from final data matrix.

### Statistical analyses

**Fig. 1:**
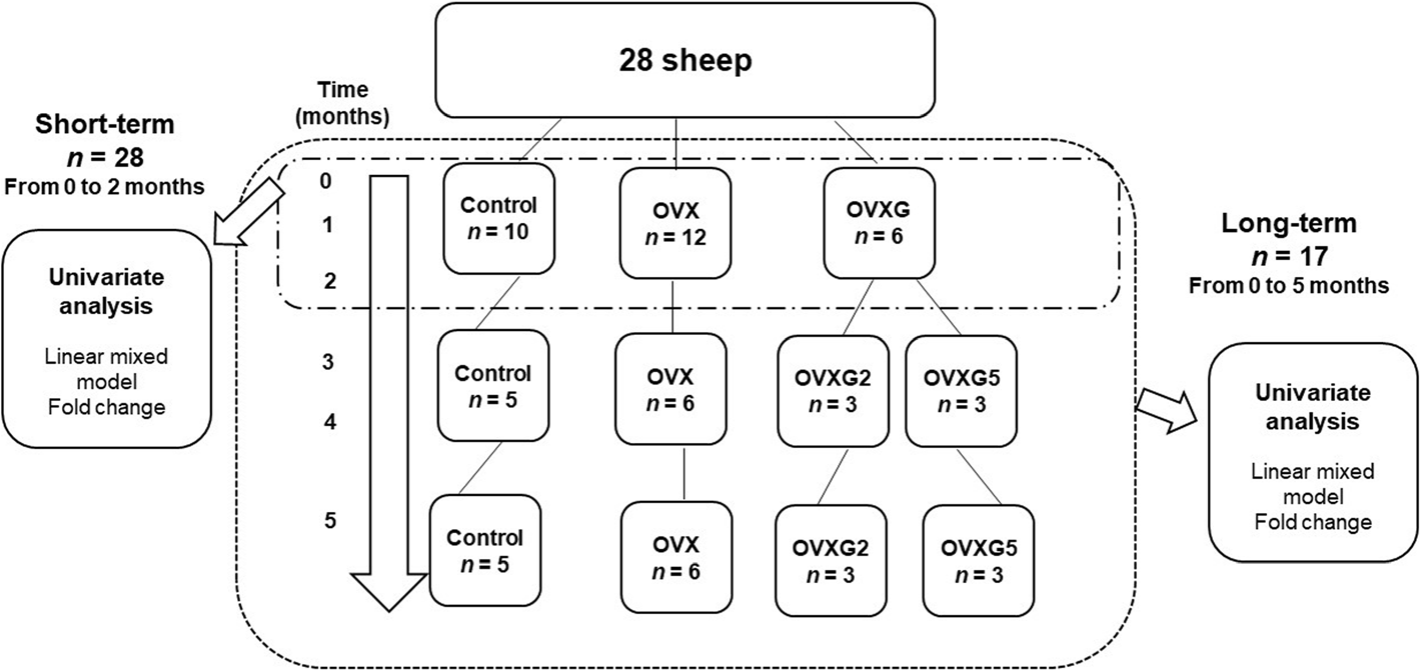
Overview of the study design and specific statistical analyses. Short-term study: Plasma metabolite and lipid profiles of 28 sheep, control group *n* = 10, OVX group *n* = 12, OVXG *n* = 6, were analyzed by multivariate and univariate analyses from baseline to two months. Long-term study: Plasma metabolite and lipid profiles of 17 sheep, control group *n* = 5, OVX group *n* = 6, OVXG2 *n* = 3, OVXG5 *n* = 3, were analyzed by multivariate and univariate analyses from baseline (month zero) to five months

The metabolite and lipid profiles obtained from LC–MS positive and negative ionization modes were analyzed by univariate analysis to assess group differences by either multivariate or univariate analysis (Fig. 1). Separate approaches were utilized to investigate the effects of short-term and long-term glucocorticoid treatment on metabolite and lipid profiles in OVX sheep. For the short-term approach, the first three-time points (0, 1 and 2 months) were analyzed for the three treatment groups (OVX, OVXG and Control; *n* = 28). For the long-term approach, all six-time points (0, 1, 2, 3, 4 and 5 months) were analyzed for the four treatment groups (OVX, OVXG, OVXG2 and Control; *n* = 17).

A linear mixed model was used to analyze for treatment effects over time in the metabolite profiles of OVX sheep. Homogeneity of group variances was estimated using Levene’s test. Changes in the sheep plasma over time were analyzed with a linear mixed effect (lme) model for comparison of means in the R environment [37], using treatment and time as fixed effects, and animal as random effect. Differences between group means of treated sheep were compared using one-way ANOVA, followed by post-hoc Fisher’s least significant difference (LSD) test for pair-wise multiple comparison of the group means. Longitudinal responses for each metabolite and lipid were reported for the following combination of groups: comparison between baseline and follow-up relative intensities of the control group; comparison between baseline and follow-up intensities of the OVX group and comparison between baseline and follow-up levels of the OVXG2 and OVG5 groups. Significance was considered at *p* < 0.05, with predicted means from the model with standard error of the means obtained using the PredictedMeans package of R. Metabolites and lipids that showed no significant differences between the interaction and treatments were not further analyzed.

## Results

For the metabolomic analysis, a total of 60 features (positive ionization) and 133 features (negative ionization) were obtained after filtration and removal of background noise and were used for the subsequent statistical analyses. 26 features were significantly affected by treatment and time in the short-term approach and 24 in the long-term approach (*p <* 0.05). Of these significant features, only nine were tentatively identified across the two-statistical analyses.

For the lipidomic analysis, 165 features (positive ionization) and 391 features (negative ionization) were obtained after filtration and removal of background noise and were fitted within the linear mixed model. 48 lipid features were significantly affected by treatment and time in the short-term approach and 126 in the long-term approach (*p <* 0.05). Of these significant features, 15 were tentatively identified across the two-statistical analyses.

### Effect of OVX and OVXG on the plasma metabolome of sheep: short-term approach

**Fig. 2:**
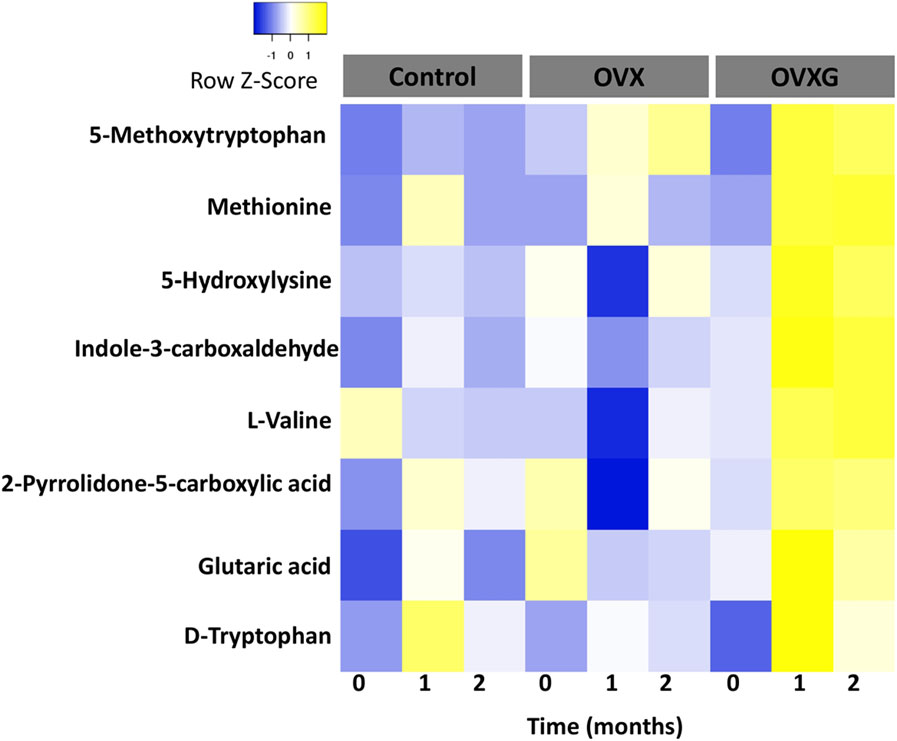
Heatmap showing the longitudinal response for each metabolite in the short-term approach. Data were calculated using a linear mixed model and mean of the relative intensities of the different treatment groups (control group (*n* = 10), OVX group (*n* = 12) and OVXG (*n* = 6)). Blue and yellow indicate decreased and increased relative intensities, respectively

To identify individual dynamic metabolite changes, all features were analyzed using a linear mixed model with 26 features significant (*p* < 0.05) and of these, eight were tentatively identified and discussed further. The relative intensities of plasma metabolites showed different longitudinal patters from baseline in all the treatment groups (Fig. 2). Changes in the plasma metabolites of the OVX group were observed with a decrease in the relative intensities of all the metabolites one month after OVX when compared with baseline, with the exception of 5-methoxytryptophan, methionine and tryptophan. However, all metabolites significantly increased in relative intensity at month two of this study, with the exception of methionine and tryptophan. In contrast, at month one, all metabolites significantly increased in relative intensity in the OVXG group when compared with baseline, while at month two, valine and glutaric acid were the only metabolite with decreased relative intensities.

The fold change (FC) highlighting the corresponding changes of metabolite profiles relative to their respective control groups (OVX vs Control and OVXG vs OVX) are also presented in Table 1. Overall, the plasma metabolite profile indicated early alterations during estrogen deficiency, and glucocorticoid treatments were significantly different between treated and control groups. Comparing OVX versus control group, only the FC values of 5-methoxytryptophan were significantly greater than 1 at month one and month two. However, comparing OVXG vs OVX, the FCs of all the metabolites were significantly greater than 1 at month one, while at month two only the fold change of methionine was greater than 2. These results suggest that OVXG resulted in a more immediate response than OVX alone.

**Table 1:** Effect of OVX and OVXG on the metabolite profile of sheep: short-term approach

*m/z*	Metabolite	OVX vs Control		OVXG vs OVX		*p*		
		FC	FC	FC	FC			
		1	2	1	2	Trt	Time	Int
233.803	5-Methoxytryptophan	**1.94**	**2.95**	**1.63**	1.20	< 0.0001	< 0.0001	< 0.0001
148.039	Methionine	0.90	1.17	**1.56**	**2.68**	0.005	< 0.0001	0.028
161.903	5-Hydroxylysine	0.79	1.11	**1.63**	1.13	0.086	0.275	0.012
144.044	Indole-3-carboxaldehyde	0.74	1.15	**2.32**	**1.73**	0.006	0.386	0.001
116.070	L-Valine	0.75	1.05	**1.75**	**1.27**	0.015	0.096	0.007
128.034	2-Pyrrolidone-5-carboxylic acid	**0.78**	1.03	**1.38**	1.08	0.445	0.4	0.009
131.081	Glutaric acid	0.89	1.15	**1.48**	1.20	0.074	0.241	0.008
203.138	D-Tryptophan	**0.73**	0.96	**1.63**	1.19	0.42	< 0.0001	0.042

Relative intensities were measured in plasma samples of control group (*n* = 10), OVX group (*n* = 12) and OVXG (*n* = 6) using linear mixed models. Results are fold changes (FC) of the lipid intensities between the OVX and control groups and between the OVXG and the OVX groups at month one and month two, among identified compounds by LC-MS. FC with a value > 1 indicated a relatively higher intensity present in treated animals, whereas a value < 1 indicated a relatively lower intensity compared with their respective control animals

All FC values, which are in bold, are significant (*p* < 0.05)

*p* calculated using post-hoc Fisher’s least significant difference (LSD) test for pair-wise multiple comparison of the group means

### Effect of OVX and OVXG on the plasma metabolome of sheep: long-term approach

**Fig. 3:**
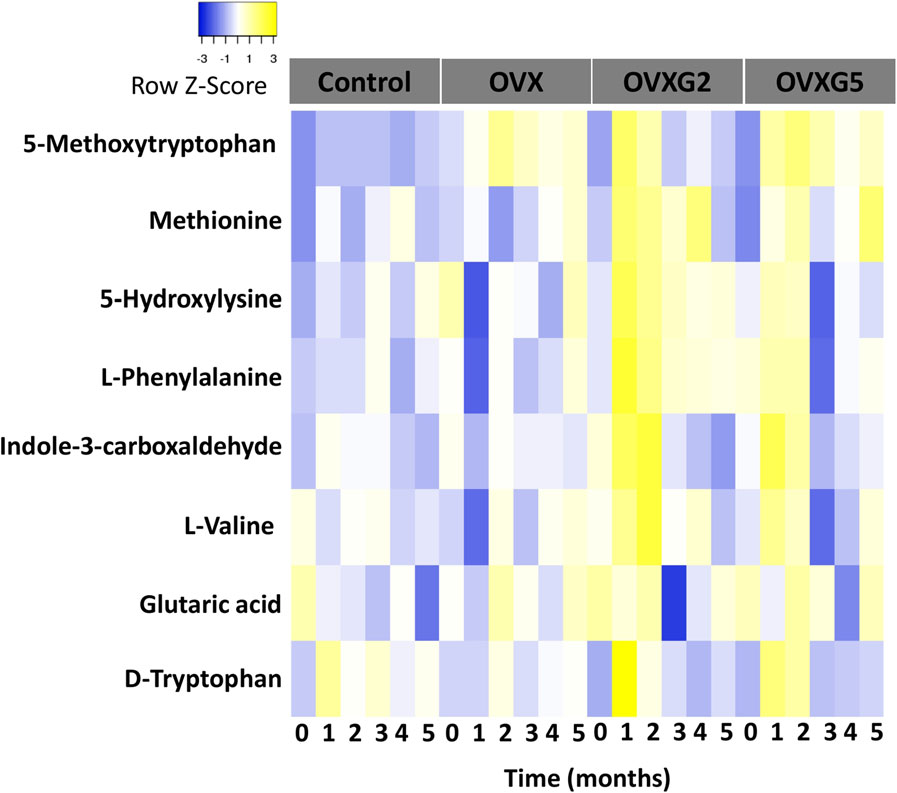
Heatmap showing the longitudinal response for each metabolite in the long-term approach. Data were calculated using a linear mixed model and mean of the relative intensities of the different treatment groups (control group (*n* = 5), OVX group (*n* = 6), OVXG2 (*n* = 3) and OVXG5 (*n* = 3)). Blue and yellow indicate decreased and increased relative intensities, respectively

Overall, the metabolites selected by the linear mixed model presented a significant interaction between treatment and time (Fig. 3). At month one, the OVX group showed increased relative intensities only for 5-methoxytryptophan and methionine when compared with baseline. In contrast, relative intensities of all metabolites apart from malic acid increased in the OVXG2 and OVXG5 groups.

At month three, in the OVX group methionine was the only metabolite with increased relative intensity. Further, the metabolite profile of OVXG2 showed a decrease in the relative intensities of all the amino acids, with the exception of methionine and phenylalanine. Similarly, all the relative intensities of the metabolites in the OVXG5 decreased.

At month five, the relative intensities of 5-methoxytryptophan, methionine, hydroxylysine, phenylalanine, valine, malic acid and tryptophan increased in the OVX group whereas the relative intensity of indole-3-carboxaldehyde decreased. Ewes in OVXG2 had decreased concentrations of 5-methoxytryptophan, methionine, phenylalanine, indole-3-carboxaldehyde and valine, and increased levels of hydroxylysine, tryptophan and malic acid. In addition, OVXG5 animals showed increased relative intensities of all amino acids, with the exception of hydroxylysine.

The FC of 5-methoxytryptophan was greater than 2 at all the time points when the OVX group was compared to the control group (Table 2). Methionine and malic acid FC values were higher than 1.5 only at month five. The OVXG5 group compared to OVX showed higher FC values than 1 for all the metabolites in month one and two. Methionine’s FC value was higher than 3 only at month two.

**Table 2:** Effect of OVX and OVXG on the metabolite profile of sheep in the long-term analysis

*m/z*	Metabolite	OVX vs Control						OVXG5 vs OVX					OVXG2 vs OVXG5				*p*		
		FC	FC	FC	FC	FC	FC	FC	FC	FC	FC	FC	FC	FC	FC	FC			
		1	2	3	4	5	1	2	3	4	5	1	2	3	4	5	Trt	Time	Int
233.803	5-Methoxytryptophan	2.11	**3.33**	**2.50**	**2.83**	**2.24**	1.39	1.09	1.08	0.92	1.06	1.31	0.80	**0.40**	0.79	0.45	0.001	< 0.0001	0.015
148.039	Methionine	0.97	0.79	0.81	0.92	**1.98**	1.18	**3.42**	1.10	0.98	1.46	1.58	1.08	1.53	1.67	**0.36**	0.098	0.003	0.012
161.903	5-Hydroxylysine	**0.68**	1.14	0.96	0.93	1.06	**1.74**	1.10	**0.67**	1.19	0.83	1.18	1.09	**1.67**	1.07	1.15	0.335	0.374	0.007
164.071	L-Phenylalanine	**0.59**	1.13	0.78	1.18	1.13	**2.22**	1.19	0.70	1.09	0.96	1.28	1.09	**1.98**	1.11	1.03	0.057	0.229	0.003
144.044	Indole-3-carboxaldehyde	**0.57**	1.06	0.97	1.27	1.52	**3.18**	1.41	0.65	0.87	1.03	0.96	1.40	1.39	0.83	0.44	0.493	< 0.0001	< 0.0001
116.070	L-Valine	0.70	1.05	0.83	1.16	1.15	**1.95**	1.07	0.74	0.82	1.01	1.00	1.26	1.59	1.29	0.79	0.418	0.136	0.011
133.043	Malic acid	0.91	1.21	1.17	0.93	**1.52**	1.10	1.02	1.03	0.82	1.03	1.09	0.98	**0.56**	1.26	0.95	0.643	0.027	0.03
203.138	D-Tryptophan	**0.57**	1.07	0.70	1.05	0.99	**1.93**	1.24	0.83	0.79	0.75	1.35	0.82	1.25	0.90	1.07	0.673	< 0.0001	0.001

Relative intensities were measured in plasma samples of control group (*n* = 5), OVX group (*n* = 6), OVXG2 (*n* = 3) and OVXG5 (*n* = 3) using a linear mixed model. Results are fold changes (FC) of the metabolite abundance between the OVX and control groups, between the OVXG5 and the OVX groups and between the OVXG2 and the OVXG5 groups from month one to month five, among identified compounds by LC-MS

FC with a value > 1 indicates a relatively higher intensity present in treated ewes, whereas a value < 1 indicates a relatively lower intensity compared with their respective control animals

All FC values in bold are significant (*p* < 0.05)

*p* calculated using post-hoc Fisher’s least significant difference (LSD) test for pair-wise multiple comparison of the group means

Similarly, the OVXG2 group compared to OVXG showed higher FC values than 1 in all the metabolites in month one, with exception of indole-3-carboxaldehyde. Notably, at month three, 5-hydroxylysine and phenylalanine increased the FC value, but 5-methoxytryptophan and malic acid were lower than 1. Further, methionine’s FC value was lower at month five.

### Effect of OVX and OVXG on the plasma lipidome of sheep: short-term approach

Forty-eight features were significant from the linear mixed model; however, only five of these were subsequently identified: one phosphatidylglycerol, two cardiolipins and two phosphatidylinositol lipids which are further described below.

**Fig. 4:**
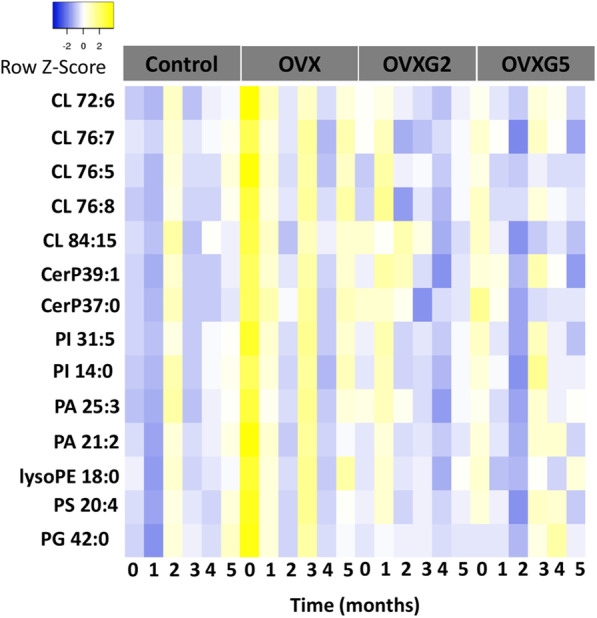
Heatmap showing the longitudinal response for each lipid in the short-term approach. Data were calculated using a linear mixed model and mean of the relative intensities of the different treatment groups (control group (*n* = 10), OVX group (*n* = 12) and OVXG (*n* = 6)). PG = phosphatidylglycerol, CL = cardiolipin, PI = phosphatidylinositol. Blue and yellow indicate decreased and increased relative intensities, respectively

The relative intensities of all the lipids at month one and month two for the OVX group decreased compared to baseline (Fig. 4). The same trend was noticed in the OVXG group at month one; however, the relative intensities of cardiolipin (CL) (76:7) and PI 14:0 increased. At month two, the relative intensities of all the lipids decreased.

The fold changes of the selected lipids changed in relative intensity of the treated groups when compared with their control groups; however, those changes were not statistically significant (Table 3). In the OVX group at month one, the relative intensity of PI species and CL (76:7) were increased by > 1.1-fold when compared with the control group. In the OVXG group, the relative intensity of all lipids decreased by < 1-fold when compared with the OVX group at month one and month two.

**Table 3:** Effect of OVX and OVXG on the lipidome of sheep in the short-term approach

*m/z*	Lipid class	OVX vs Control		OVXG vs OVX		*p*		
		FC	FC	FC	FC			
		1	2	1	2	Trt	Time	Int
735.521	PG 33:0	1.08	0.91	1.01	1.01	0.002	0.075	0.003
752.511	CL 76:7	1.11	1.00	1.01	0.83	0.004	0.003	0.013
726.496	CL 72:5	1.08	1.02	1.00	0.96	0.005	0.011	0.016
785.429	PI 31:5	1.15	0.98	0.99	0.87	0.011	0.01	0.028
557.236	PI 14:0	1.23	0.94	1.02	0.82	0.035	0.104	0.038

Relative intensities were measured in plasma samples of control group (n = 10), OVX group (n = 12) and OVXG (n = 6) using linear mixed models. Results are fold changes (FC) of the lipid intensities between the OVX and control groups and between the OVXG and the OVX groups at month one and month two, among identified compounds by LC-MS. FC with a value > 1 indicated a relatively higher intensity present in treated animals, whereas a value < 1 indicated a relatively lower intensity compared with their respective control animals

*PG* phosphatidylglycerol, *CL* cardiolipin, *PI* phosphatidylinositol

### Effect of OVX and OVXG on the plasma lipidome of sheep: long-term approach

**Fig. 5:**
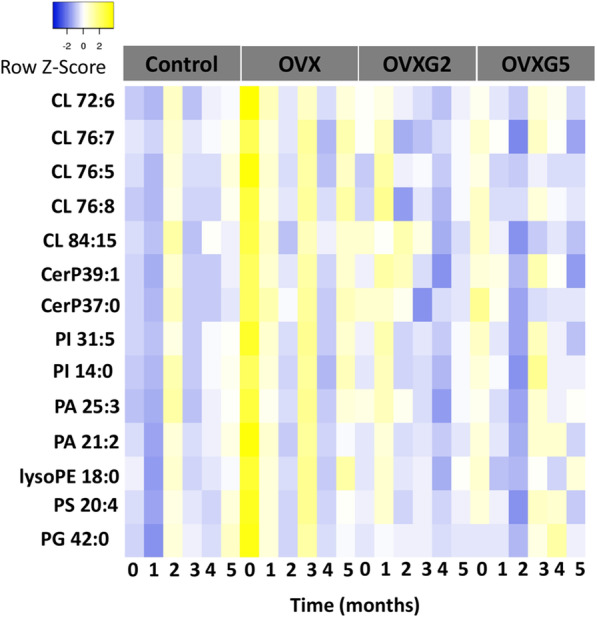
Heatmap showing the longitudinal response for each lipid in the long-term approach. Data were calculated using a linear mixed model and mean of the relative intensities of the different treatment groups (control group (*n* = 5), OVX group (*n* = 6), OVXG2 (*n* = 3) and OVXG5 (*n* = 3). CL = cardiolipin, CerP = ceramide 1-phosphates, PI = phosphatidylinositol, PA = phosphatidic acid, lysoPE = lysophosphatidylethanolamines, PS = phosphatidylserines, PG = phosphatidylglycerol. Blue and yellow indicate decreased and increased relative intensities, respectively

126 features were significant from the interaction between treatment and time; however, only 14 of these features were subsequently identified (Fig. 5). At month one, the OVX group showed decreased relative intensities of all lipids when compared with baseline. In the OVXG2 group all the lipids increased relative intensities, with exception of CL (84:15). While, in the OVXG5 group, PG (42:0) was the only lipid with increased relative intensity. By contrast, the OVX and OVXG5 groups showed increased relative intensities of all the lipids at month three. However, in the OVXG2 group, increased relative intensities of three CL species (76:7, 72:5, 76:8), two PI species (31:5, 14:0), PA (21:2), lysoPE and PS were observed.

At month five, increased relative intensities of all the lipids in the OVX and OVXG2 groups were observed, while in the OVXG5 group, all the peak intensities of lipids were decreased, with exception of PA (25:3) and lysoPE.

The OVX compared to control group showed a significant FC of four lipids at month one, three at month two and eleven at month three (significant lipids presented with bolded FC values, *p* < 0.05, Table 4). All the lipids at month one, month three and month five (with the exception PA (21:2), PS and PG) were increased in relative intensities by > 1-fold. A significant increase of FC values was noticed at month one in CL (84:15), CerP species and PG; at month two in CL (84:15), PI (14:0) and PA (25:3); and at month three in CL (76:7, 72:5, 76:8, 84:15), CerP species, PI species, PA (25:3), lysoPE and PS.

**Table 4:** Effect of OVX and OVXG on the lipidome of sheep in the long-term analysis

*m/z*	Lipid class	OVX vs Control						OVXG5 vs OVX					OVXG2 vs OVXG5				*p*		
		FC	FC	FC	FC	FC	FC	FC	FC	FC	FC	FC	FC	FC	FC	FC			
		1	2	3	4	5	1	2	3	4	5	1	2	3	4	5	Trt	Time	Int
725.492	CL 72:6	1.20	0.91	1.16	0.98	1.05	0.89	0.96	0.99	1.06	0.90	1.06	1.05	0.91	0.90	1.04	0.018	0.082	0.023
752.511	CL 76:7	1.15	0.89	**1.21**	0.87	1.11	0.95	0.82	0.94	1.15	**0.75**	1.10	1.09	0.82	0.94	1.17	0.088	0.023	0.016
726.496	CL 72:5	1.19	0.91	**1.19**	0.95	1.02	0.88	0.98	0.87	1.06	0.88	1.19	1.05	1.01	0.97	1.05	0.03	0.026	0.018
751.518	CL 76:8	1.23	0.92	**1.26**	1.02	1.12	0.88	0.98	0.92	1.07	0.84	1.27	0.87	0.90	0.86	1.03	0.011	0.06	0.042
800.511	CL 84:15	**1.15**	**0.85**	**1.16**	0.97	1.07	0.92	0.93	**0.87**	0.99	0.88	1.03	**1.26**	1.10	0.93	1.04	0.027	0.06	0.005
686.546	CerP 39:1	**1.21**	0.91	**1.21**	1.02	1.09	0.97	0.93	0.99	1.08	**0.80**	1.08	1.22	0.86	0.83	1.13	0.042	0.071	0.014
660.543	CerP 37:0	**1.17**	0.93	**1.16**	0.99	1.10	0.94	0.91	**0.88**	1.04	0.92	1.04	1.13	0.91	0.99	1.00	0.019	0.029	0.045
785.429	PI 31:5	1.31	0.87	**1.42**	0.90	1.09	0.85	0.85	0.93	1.11	0.77	1.24	1.13	0.82	0.89	1.18	0.022	0.022	0.016
557.236	PI 14:0	1.43	**0.73**	**1.52**	0.81	1.14	0.89	0.76	1.02	1.20	0.80	1.20	1.30	0.69	0.83	1.03	0.12	0.153	0.019
543.316	PA 25:3	1.23	**0.81**	**1.33**	0.92	1.06	0.92	0.89	0.91	1.09	0.94	1.19	1.25	0.88	0.83	0.99	0.117	0.103	0.018
489.253	PA 21:2	1.40	0.79	1.31	0.94	0.93	0.86	0.94	0.90	1.24	0.90	1.13	1.13	0.86	0.79	1.04	0.099	0.046	0.024
464.323	lysoPE 18:0	1.44	0.83	**1.41**	0.93	1.19	0.77	0.88	0.82	1.04	0.92	1.33	1.16	0.95	0.90	0.93	0.014	0.004	0.043
558.243	PS 20:4	1.36	0.85	**1.32**	1.02	0.93	0.90	0.83	0.91	1.22	0.88	1.12	1.20	0.86	0.80	1.09	0.073	0.036	0.038
861.656	PG 42:0	**1.39**	0.84	1.22	1.00	0.89	0.89	0.93	0.90	1.26	0.98	1.03	1.16	0.92	0.80	0.99	0.138	0.185	0.026

Relative intensities were measured in plasma samples of control group (*n* = 5), OVX group (*n* = 6), OVXG2 (*n* = 3) and OVXG5 (*n* = 3) using a linear mixed model. Results are fold changes (FC) of the lipid abundance between the OVXG5 and the OVX groups and between the OVXG2 and the OVXG5 groups from month one to month five, among identified compounds by LC-MS. FC with a value > 1 indicated a relatively higher intensity present in treated animals, whereas a value < 1 indicated a relatively lower intensity compared with their respective control animals

All FC values, which are in bold, are significant at *p* < 0.05 calculated using post-hoc Fisher’s Least Significant Difference (LSD) test for pair-wise multiple comparison of the group means

*CL* cardiolipin, *CerP* ceramide 1-phosphates, *PI* phosphatidylinositol, *PA* phosphatidic acid, *lysoPE* lysophosphatidylethanolamines, *PS* phosphatidylserines, *PG* phosphatidylglycerol

Combination treatments in the OVXG group induced an increase in relative intensities of lipids by > 1-fold at month four when compared with the OVX group (with exception of CL (84:15). A significant decreased of FC values was noticed at month three in CL (84:15) and CerP (37:0); and month five in CL (76:7) and CerP (39:1). In the OVXG2 group the FC values were higher than 1 in month one; month two with exception of CL (76:8); and month five with exception of PA (25:3), lysoPE and PG. CL (84:15) was significantly increased at month two when compared with the OVXG group. These results suggest that OVX or the combination of OVX and glucocorticoid treatments affected the lipid profiles of sheep by inducing changes in lipid metabolism.

## Discussion

In this pilot study we describe LC–MS metabolomics and lipidomics approaches to identify changes in the plasma metabolome and lipidome of OVX and OVXG sheep as a large animal model for postmenopausal osteoporosis. This is the first longitudinal study that presents results on the plasma metabolite and lipid profiles of OVX sheep treated with glucocorticoids as an animal model of osteoporosis. The results obtained demonstrate metabolite and lipid changes in response to hormonal interventions over time: either OVX alone or OVX in combination with glucocorticoid treatments. Results of both the short-term and long-term statistical analysis approach from the linear mixed models highlights mass spectral features that change over the two- or five-month study period.

### Effect of OVX and OVXG on the plasma Metabolome of sheep

Osteoporosis in this model was associated with early changes in the plasma metabolite profile, including amino acids and other metabolites. Overall, circulating metabolites were elevated in plasma after OVX in sheep. In this study, compared to the OVX animals, OVXG2 animals could reverse the impacts of glucocorticoids on the plasma metabolites at month four and month five. These results suggest that short-term administration of glucocorticoid might be reversible as the relative intensities of the metabolites in the OVXG2 group showed a slight return on the abundance levels similar to those ones observed in the OVX group. Further, in the OVXG5 group longer administration of glucocorticoid treatment altered their metabolite profile. The OVX sheep is an established model for osteoporosis [38]. The changes observed in the metabolite profile suggested that OVX alone or when combined with glucocorticoid treatments affects plasma metabolites during the progression of postmenopausal osteoporosis in sheep. The dynamic change observed in the plasma metabolite profile of the OVX sheep may reflect systemic changes related to the increased bone turnover observed in estrogen deficiency models, where an imbalance within the bone remodelling process suggests enhanced bone resorption and a reduced bone formation [32]. In addition, due to uncoupling of the bone turnover cycle in the presence of low estrogen, bone resorption increases at basic multicellular units of bone, and the rate of bone loss is greater than the formation of new bone [39, 40]. In the glucocorticoid-induced osteoporosis model, bone resorption markers have been associated with a decreased BMD as a result of an excessive bone resorption rate and reduced osteoblast activity [41].

Amino acids are not only the building blocks of proteins but are also substrates for secondary metabolism and are involved in the process of cell signalling and signal transduction of key metabolic pathways. Amino acids enhance immunity by upregulating pro-inflammatory and downregulating anti-inflammatory cytokines [42]. Menopause is associated with the risk of developing several chronic diseases including cardiovascular disease, diabetes and osteoporosis, among others. Osteoporosis is accompanied by oxidative stress and inflammation, and changes of amino acid levels may indicate which key metabolic pathways may be altered in postmenopausal women [43–46].

Although this is the first study that reports changes in the plasma metabolite profile of OVX and OVXG sheep, previous metabolomic analyses have shown altered metabolism in OVX rats, another common animal model for postmenopausal osteoporosis. A previous study using nuclear magnetic resonance reported that six months after OVX, serum levels of alanine, asparagine, glutamine, isoleucine, leucine, valine, tryptophan, lysine, proline, serine and threonine increased, and glutamate, glycine, phenylalanine and tyrosine were decreased [47]. Another study reported increased serum concentrations of isoleucine, valine and leucine after OVX using gas chromatography time-of-flight mass spectrometry [48]. Similarly, branched-chain amino acids (valine, leucine and isoleucine), homocysteine, hydroxyproline and ketone bodies (3-hydroxybutyric acid) have been observed to be elevated, while levels of lysine decreased in the OVX group compared to the sham group [17].

In this study, plasma methionine levels were increased in the OVXG group. Methionine plays key roles in cell metabolism as one of the essential amino acids required for cellular proliferation, protein synthesis, RNA synthesis and transamination. Further, methionine can be catabolized into homocysteine and cysteine [49, 50]. A previous study on methionine reported changes in bone turnover in hyperhomocysteinemia aged rats, where osteocalcin (OC) decreased and urine N-terminal type 1 collagen, a marker of bone resorption, increased [51]. Hermann et al. [52] reported a positive correlation between hyperhomocysteinemia and urinary deoxypyridinoline crosslinks in peri- and postmenopausal women; however, no significant correlations were observed between hyperhomocysteinemia and OC. In another study, hyperhomocysteinemia levels were associated with higher bone turnover and lower BMD [53]. This suggests that circulating levels of methionine may alter bone remodelling and could possible explain the reduced BMD observed in treated ewes by upregulating osteoclast development induced by OVX and glucocorticoid treatments.

In this study, tryptophan levels were increased in the OVX group. Tryptophan and its metabolites impact bone formation, stimulating the proliferation and differentiation of bone marrow-derived mesenchymal stem cells [54]. The possible mechanism of altered tryptophan in bone loss may be linked to enhanced osteoclastogenesis. Studies have indicated that tryptophan may play a role in osteoclastogenesis via the kynurenine pathway of tryptophan catabolism, as interferon *y* (IFN-*y*) is formed via the kynurenine pathway [55]. IFN-*y* is a cytokine that is produced in the bone microenvironment by cells, and it promotes osteoclast activity. During menopause, estrogen deficiency upregulates the pro-inflammatory cytokines, as IFN-*y* increases the bone loss in postmenopausal women [56, 57]. The results in this study provide evidence that OVX and glucocorticoid treatment perturbed plasma tryptophan levels in both OVX and OVXG sheep. Further studies in animal models are needed to determine whether tryptophan metabolites could be utilized as novel biomarkers for bone loss in human osteoporosis.

Plasma concentrations of hydroxylysine were decreased in the OVXG group when compared with the OVX control group. Hydroxylysine is a urinary marker of bone resorption derived from collagen crosslinks [58]. In bone tissue, bone collagen proteins comprise about 90% of the organic matrix of the bone. Bone collagen supports properties of bone such as tissue mass, micro- and macro-architecture and material properties, which all contribute to bone strength [59]. Bone collagen is rich in proline, hydroxyproline and glycine. Proline and lysine residues are hydroxylated to hydroxyproline and hydroxylysine, respectively, which contribute to the formation of collagen crosslinks and to bone matrix quality [60–62]. Circulating levels of hydroxylysine as biomarker have been reported in human osteoporotic bone matrix [63]. Although the hydroxylysine urinary marker was not measured here, an decreased in the bone resorption marker C-terminal telopeptide of type I collagen was seen in the OVXG sheep plasma [32]. Thus, the plasma levels of hydroxylysine in OVXG sheep may reflect the increased bone remodelling as a response to estrogen deficiency and glucocorticoid intervention. Functional studies will be required to determine whether the identified metabolites had a direct effect on bone remodelling.

### Effect of OVX and OVXG on the plasma Lipidome of sheep

The results revealed differences in the lipidome in both short-term and long-term approaches, which indicates effects on circulating plasma lipids. The dynamic changes of the lipidome of OVX sheep, along with OVXG2 and OVXG5 were evaluated.

The short-term statistical approach revealed OVX increased CL (76:7) and PI species at month one. In contrast, in the OVXG group, a decrease in levels of all identified lipids was observed at month one, and the same effect was observed at month two. In general, this study had a small sample size for a lipidomic analysis; however, the short-term approach provided a better probability to improve the characterization of the lipidome in OVX sheep due to it having more animals in each treatment group (control group *n* = 10; OVX *n* = 12; and OVXG *n* = 6).

The long-term statistical approach highlighted lipid profile changes after OVX or OVXG2/OVXG5 treatments. Relative intensities of lipids were increased at months one and three and remained relatively stable until the end of the study in the OVX group. In contrast, in the OVXG2, a dynamic lipid compositional change could be observed from month one. These relative intensities decreased steadily until the end of this study, with the exception of CL (72:6), CerP (39:1) and PI (31:5); while in the OVXG5 treatment, the relative intensities of the all lipids decreased after five months.

The OVX sheep model was selected because it mimics postmenopausal bone loss and it is recommended by the WHO as a large species for evaluating skeletal osteoporosis therapies [64]. Lipids are involved in several cellular functions and physiological conditions associated with energy storage, structure, apoptosis and signalling, and these have an effect on skeletal metabolism and bone health [65]. In this study, lipid profiles were affected, with various relative intensities of lipids changing during the early stage of estrogen withdrawal in the OVX sheep. Although basic lipid clinical markers such as triglycerides, total cholesterol, low density lipoprotein cholesterol or high-density lipoprotein cholesterol were not measured, these findings coincide with previous results, where it was reported that perturbed levels of lipid metabolism are associated with osteoporosis [41, 66–68]. Further studies are required to validate the findings presented in this study; however, the lipids identified provide an overview of the lipidomic changes during postmenopausal osteoporosis in OVX sheep.

In this study, levels of phospholipids decreased after OVX and increased at month three until the end of the study. Although this is the first study that presents lipid profiles of OVX sheep, previous studies have reported lipidome changes in OVX rats. Zhu, Liu [69] reported decreased eicosapentaenoic acid, ergocalciferol and cholecalciferol, and increased arachidonic acid, in OVX rats. Conversely, an earlier study reported increased levels of fatty acids (arachidonic acid and octadecadienoic acid) and cholesterol after OVX compared to before surgery [17, 70]. However, in this study, arachidonic acid was not detected; PCs and PIs are substrates for this fatty acid and may be involved in the bone loss in the OVX sheep [71]. This implies that altered phospholipids might be involved in osteoclastogenesis promoting loss of BMD, which affects bone remodelling as result of estrogen depletion.

Sphingolipids are a major component of cell membranes and play a role in cell signalling. Ceramides are precursors to many sphingolipids. In blood plasma, they are associated with lipoproteins [72, 73]. The major subclass identified in this study was two species of ceramide phosphate. Our findings were similar to a previous study that reported that levels of ceramide, ceramide-1-phosphate, sphingomyelin, 1-*O*-alkenyl-lysophosphatidylethanolamine and lysophosphatidylethanolamine were elevated in the OVX rats compared to those in the sham-operated rats [74]. Ceramides have been implicated in increasing the cellular oxidative state, and this has been linked to stress or death signals. Ceramide levels have been reported to affect osteoblast apoptosis [75, 76]. Thus, perturbed ceramide levels may play a key role in bone loss in postmenopausal women.

Cardiolipins (CL) are phospholipids that are embedded in the inner mitochondrial membrane and are a key factor for energy production. Current evidence highlights that mitochondrial dysfunction by reactive oxygen species leads to the oxidation of mitochondrial cardiolipins, and this has been linked to atherosclerosis [77, 78]. Although the results presented here are unable to establish a direct role of CL in osteoporosis, it may be related via bone marrow cells, where estrogen withdrawal regulates the complex cell differentiation of osteoblasts and adipocytes [79].

The data reported here is the outcome from a liner mixed model of a pilot intervention, where we analyzed both the metabolome and lipidome data without adjusting for baseline values. The longitudinal responses for each metabolite and lipid were measured and reported for the same group of ewes repeatedly as the goal was to understand the metabolic and lipidomic alterations that accompany bone loss in osteoporosis development. The result from the linear mixed model specifically indicated no significant different between the baseline group means for the metabolome results. However, for the baseline lipidome results significant differences between-group were noted. In metabolomics studies, both intrinsic and extrinsic factors are responsible for changes occurring in the metabolome and lipidome of biological samples, and therefore the pre-analytical operation procedure including general experimental design and blood collection require careful sample handing to ensure a reproducible extraction is performed. In this pilot study, the ewes (7–9 years) were first fed a basal diet for 10 days (adaption period), then randomly allocated to four treatment groups with limited study size due to some operation constraints. Therefore, it must be noted that the effects reported here may be influenced by individual variation within the OVX sheep and the findings presented should be considered as baseline for follow-up studies.

Strengths of this study are the application of untargeted metabolite profiling using HILIC–MS and lipidomic analysis of plasma samples from OVX sheep to explore the dynamics of disease progression in the metabolite profile, and subsequent identification of plasma biomarkers of osteoporosis. Limitations of this study include the small sample size of treated groups, specifically the ewes that were treated with glucocorticoids, and due to the large variability of the data further validation will be required. In addition, blood samples were collected once a month, as the study design considered both a short- and long-term response to treatments. Larger sample size, and multiple sampling time points from the early stage of estrogen deficiency and glucocorticoid treatment, should be considered in future studies to identify biomarkers that are unique to the early onset of osteoporosis in postmenopausal women. Finally, although a large number of features resulted significant from the interaction treatment and time from the linear mixed model, most of the features remain unidentified. Further, the analysis of other biological matrices such as bone tissues would maximize the identification of molecular changes of the bone microenvironment in osteoporosis. Follow-up studies also should be conducted to examine the effects on the metabolome and lipidome of the microbiome and a low calcium and vitamin D diet on osteoporosis prevention.

## Conclusions

The findings of this pilot study highlighted that OVX alone or when combined with glucocorticoid treatments altered plasma metabolite and lipid profiles in sheep. The results suggest that the biosynthesis of phenylalanine, tyrosine and tryptophan, and the metabolism of cysteine, methionine and the branched-chain amino acids, valine, leucine and isoleucine, are potentially the main perturbed metabolic pathways regulating bone loss in OVX sheep. Differences observed in the relative intensities of the lipid classes CL, PI, PA and PS after OVX relate to perturbations in glycerophospholipid and phosphatidylinositol pathways. The measurement of changes in circulating metabolites and lipids might have potential implications for identification of further risk factors associated with estrogen withdrawal in women that may contribute to loss of BMD. These findings suggest new directions to examine the mechanisms of bone loss in OVX sheep and indicate that these specific metabolites could have value as prognostic biomarkers for osteoporosis in humans.

## Supplementary information

**Additional file 1: Table S1.** XCMS main parameters applied for untargeted LC-MS spectral processing.

## Data Availability

The datasets used during the current study are available from the corresponding author on reasonable request.

## Abbreviations

ACN: Acetronitrile
BMD: Bone mineral density
CL: Cardiolipin
CTx-1: C-terminal telopeptide of type 1 collagen
CerP: Ceramide-1-phosphate
DG: Diacylglycerol
DXA: Dual x-ray absorptiometry
EDTA: Ethylenediamine tetraacetic acid
HPLC: High-performance liquid chromatography
LC-MS: Liquid chromatography−mass spectrometry
LSD: Least significant difference
lysoPE: Lysophosphatidylethanolamines
OPLS: Orthogonal partial least squares
OVX: Ovariectomy
OVXG5: Ovariectomized plus five doses of glucocorticoids
OVXG2: Ovariectomized plus two doses of glucocorticoids
PA: Phosphatidic acid
PI: Phosphatidylinositol
PG: Phosphatidyglycerol
Plasmenyl-PE: Plasmenylphosphatidylethanolamine
PS: Phosphatidylserine
QC: Quality control
S1P: Sphingosine-1-phosphate
SC: Singaporean–Chinese
TCA: Tricarboxylic acid
TG: Triacylglycerol
