# Correction to: Effects of short- and long-term glucocorticoid-induced osteoporosis on plasma metabolome and lipidome of ovariectomized sheep

**DOI:** 10.1186/s12891-020-03485-x

**Published:** 2020-07-29

**Authors:** Diana Cabrera, Marlena Kruger, Frances M. Wolber, Nicole C. Roy, Karl Fraser

**Affiliations:** 1 Food Nutrition & Health Team, AgResearch Grasslands, Tennent Drive, Palmerston North, 4442 New Zealand; 2 School of Health Sciences, Massey University, Tennent Drive, Palmerston North, 4442 New Zealand; 3 Riddet Institute, Massey University, Palmerston North, 4442 New Zealand; 4 School of Food Advanced technology, Massey University, Tennent Drive, Palmerston North, 4442 New Zealand; 5 Centre for Metabolic Health Research, Massey University, Tennent Drive, Palmerston North, 4442 New Zealand; 6 High-Value Nutrition National Science Challenge, Auckland, 1142 New Zealand

**Correction to: BMC Musculoskelet Disord (2020) 21:349 **

**https://doi.org/10.1186/s12891-020-03362-7**

Following publication of the original article [1], the authors noticed that incorrect Fig. 4 was published.

**Fig. 4:**
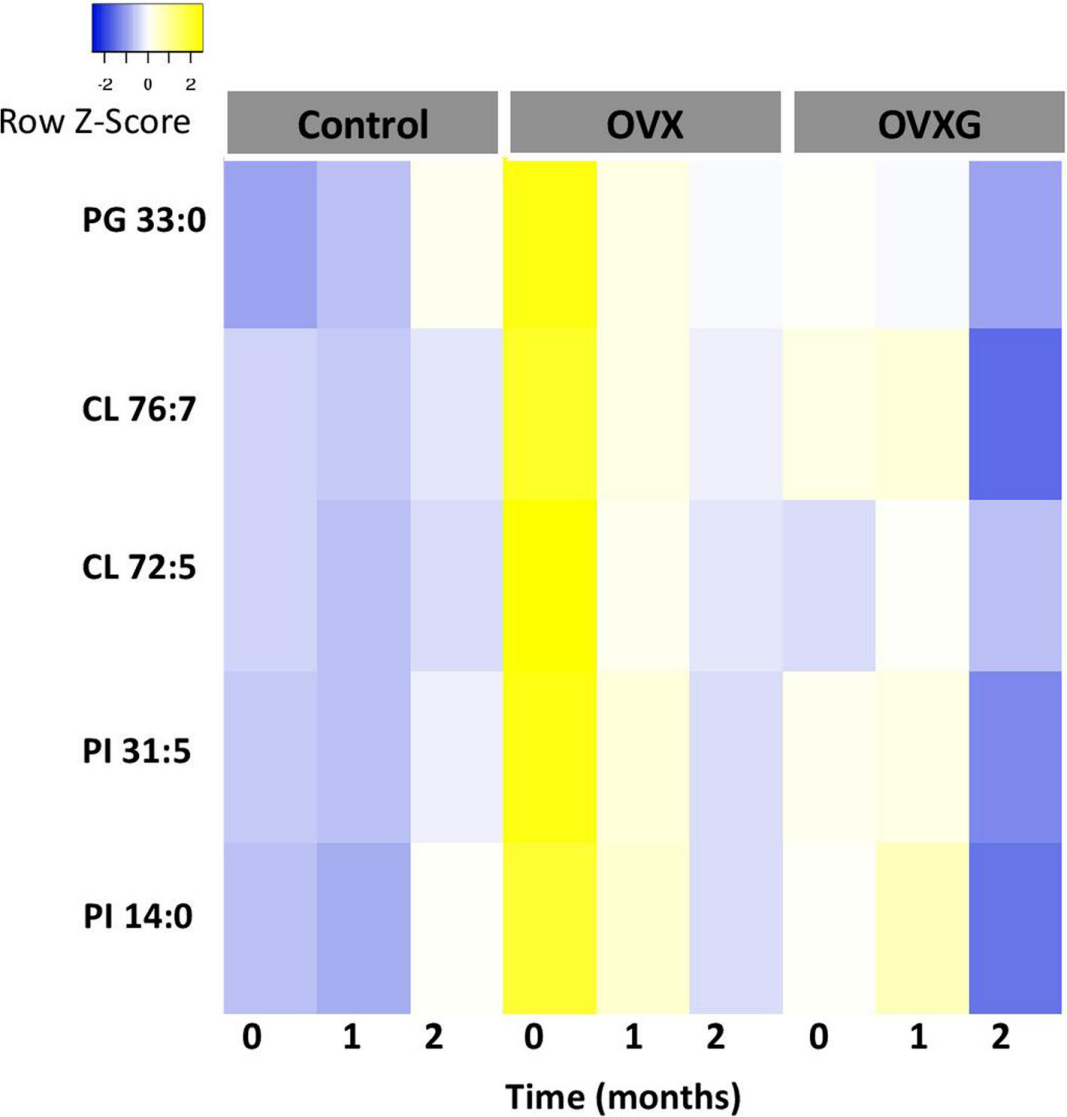
Heatmap showing the longitudinal response for each lipid in the short-term approach. Data were calculated using a linear mixed model and mean of the relative intensities of the different treatment groups (control group (*n* = 10), OVX group (*n* = 12) and OVXG (*n* = 6)). PG = phosphatidylglycerol, CL = cardiolipin, PI = phosphatidylinositol. Blue and yellow indicate decreased and increased relative intensities, respectively

The Fig. 4 is a heatmap showing the longitudinal response for each lipid in the short-term approach; however the short term approach involves only the control group, OVX group and OVXG and three time points (0, 1 and 2 months).

The correct Fig. 4 is shown below.
