# Supplementary material for: Effects of short- and long-term glucocorticoid-induced osteoporosis on plasma metabolome and lipidome of ovariectomized sheep
Source: BMC Musculoskelet Disord. 2020 Jun 5;21:349. doi: 10.1186/s12891-020-03362-7 (PMC7275480; doi:10.1186/s12891-020-03362-7)
Supplement: Supplementary file 1 — Additional file 1: Table S1. XCMS main parameters applied for untargeted LC-MS spectral processing. [file 12891_2020_3362_MOESM1_ESM.docx]

S1. XCMS main parameters applied for untargeted LC-MS spectral processing

| Parameters | Related to | Settings for metabolomics | Settings for lipidomics |
| --- | --- | --- | --- |
| ppm | *m/z* | 10 | 10 |
| peakwidth | retention time | 20,60 | 5,20 |
| Prefilter | intensity | 3,1000 | 3,20000 |
| Snthresh* | intensity | 10 | 20 |
| noise | intensity | 200 | 10000 |
